# Supplementary material for: AnnoTree: visualization and exploration of a functionally annotated microbial tree of life
Source: Nucleic Acids Res. 2019 Apr 9;47(9):4442–8. doi: 10.1093/nar/gkz246 (PMC6511854; doi:10.1093/nar/gkz246)
Supplement: Supplementary Data [file gkz246_supplemental_files.zip › Supplement.pdf]

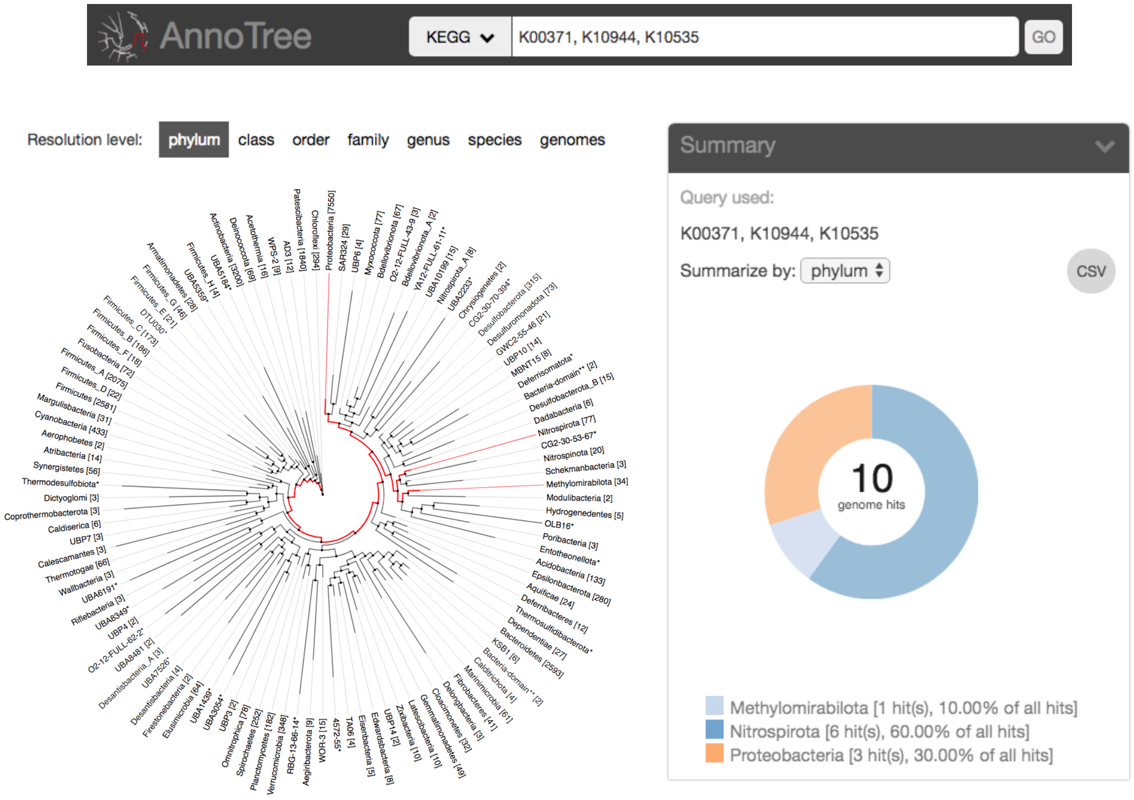

Supplemental Figure S1. Example AnnoTree search for commamox organisms using a combination query. AnnoTree was queried for genomes possessing all of three genes: *hao* (K10535), *nxrB* (K00371), and *amoA* (K10944). The results were visualized at the phylum level.

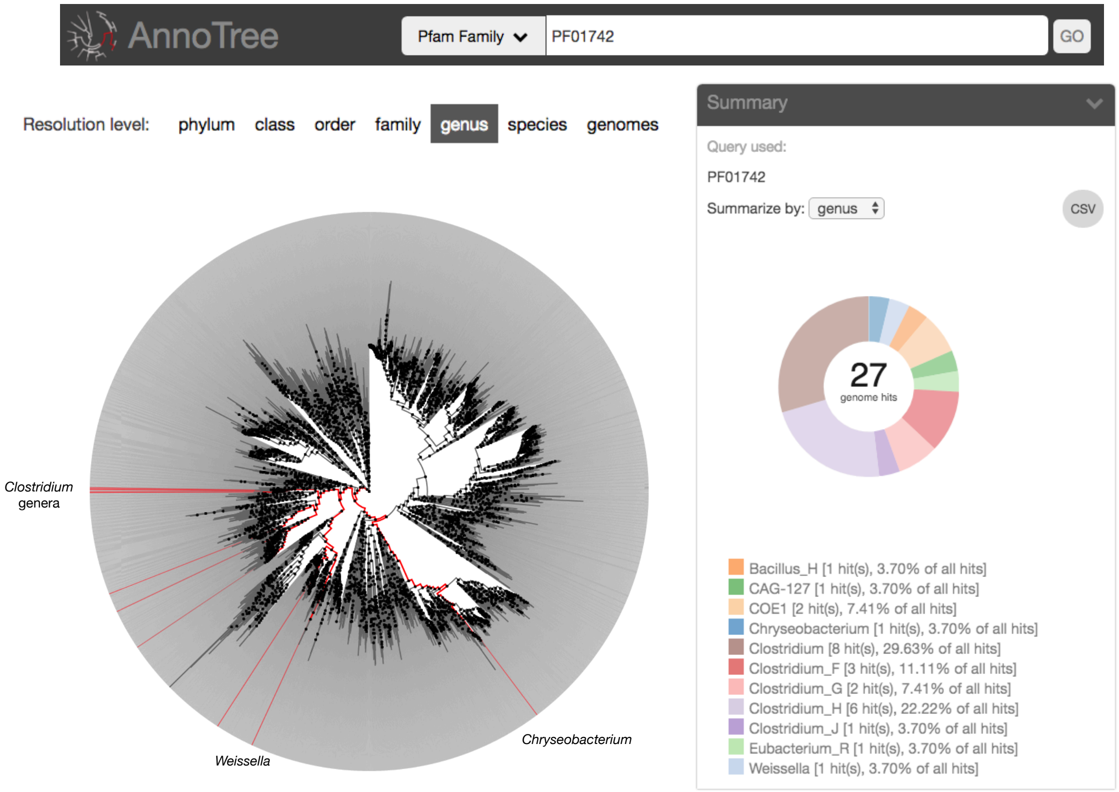

Supplemental Figure S2. Example AnnoTree search for organisms containing botulinum-neurotoxin like proteins using Pfam domain PF01742 (botulinum neurotoxin peptidase M27 domain). The results were viewed at the global genus level.

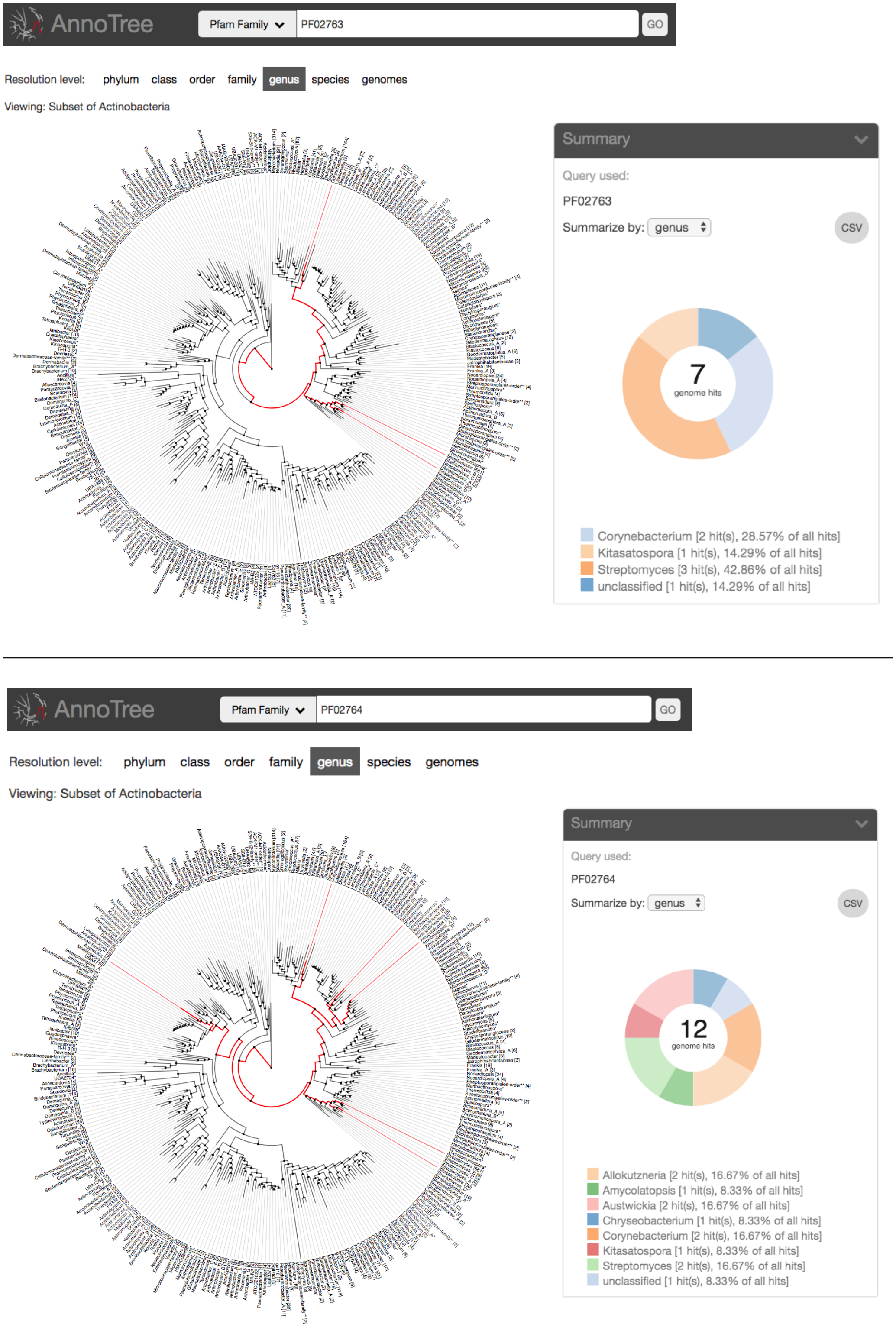

Supplemental Figure S3. Example AnnoTree search for organisms containing putative diphtheria-like toxin genes using Pfam queries PF02763 (Diphtheria_C) and PF02764 (Diphtheria_T domain). Both results were viewed at the genus level within a subset of the Actinobacteria.

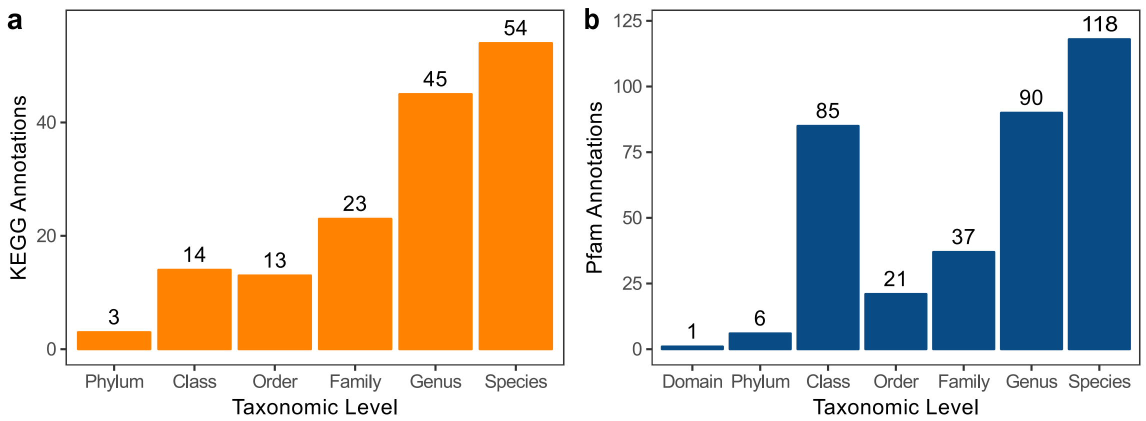

Supplemental Figure S4. Frequency of lineage-specific annotations classified at each taxonomic level. Internal nodes of the GTDB tree were classified as lineage-specific for a trait if at least 95% of the genomes containing the trait were contained within the clade, at least 95% of the genomes in the clade had the trait, and the trait was present in no more than half of the genomes in the GTDB tree. The taxonomic level of each root of a lineage-specific node was classified as the lowest common taxonomic rank between all encompassing genomes. The number of lineage-specific KEGG KO annotations (**a**) and Pfam protein families (**b**) are displayed in decreasing taxonomic order. The abundance of lineage-specific Pfam annotations at the class level is due to numerous photosynthesis-related protein families unique to the Oxyphotobacteria.

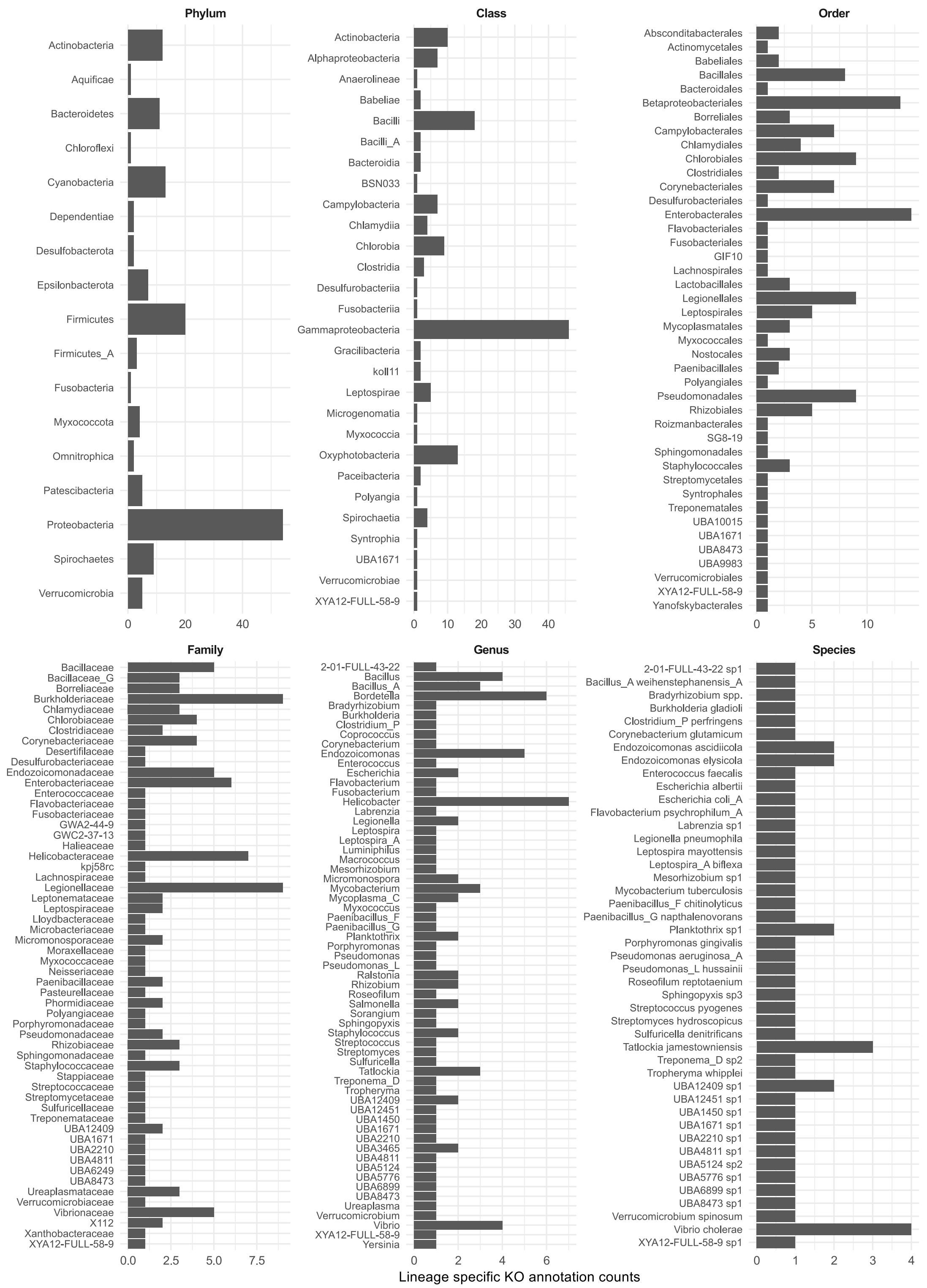

Supplemental Figure S5. Taxonomic distribution of lineage-specific KO annotations. The taxonomic identity of each lineage-specific KO annotation was determined and counted at each level. Counts at higher levels include all lineage-specific traits at that level and all encompassing lower levels.

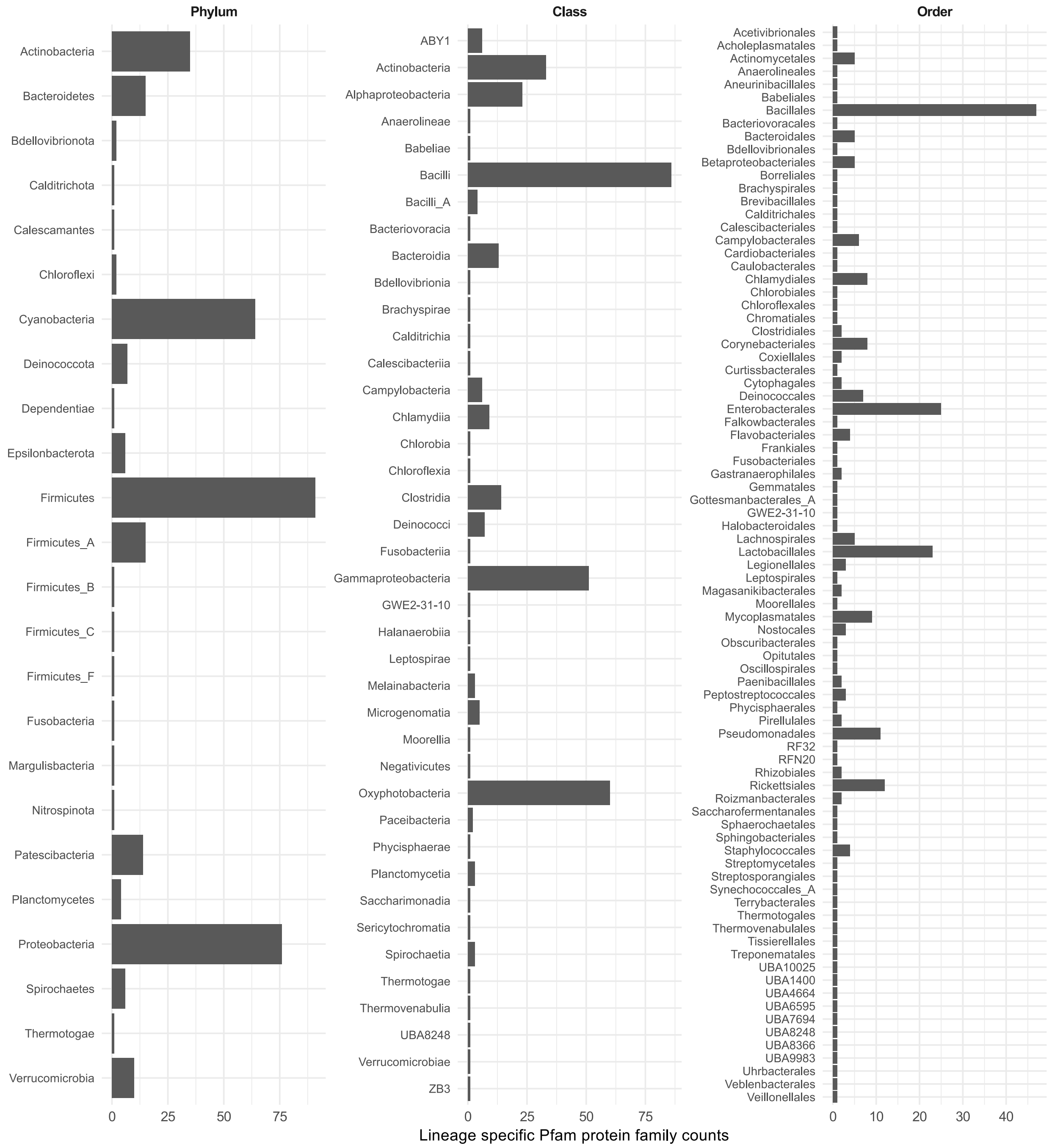

Supplemental Figure S6. Taxonomic distribution of lineage-specific Pfam protein families in higher levels. The taxonomic identity of each lineage-specific Pfam protein family was determined and counted at each level. Counts at higher levels include all lineage-specific traits at that level and all encompassing lower levels. See **Supplemental Figure 7** for Pfam protein family counts at the family, genus, and species levels.

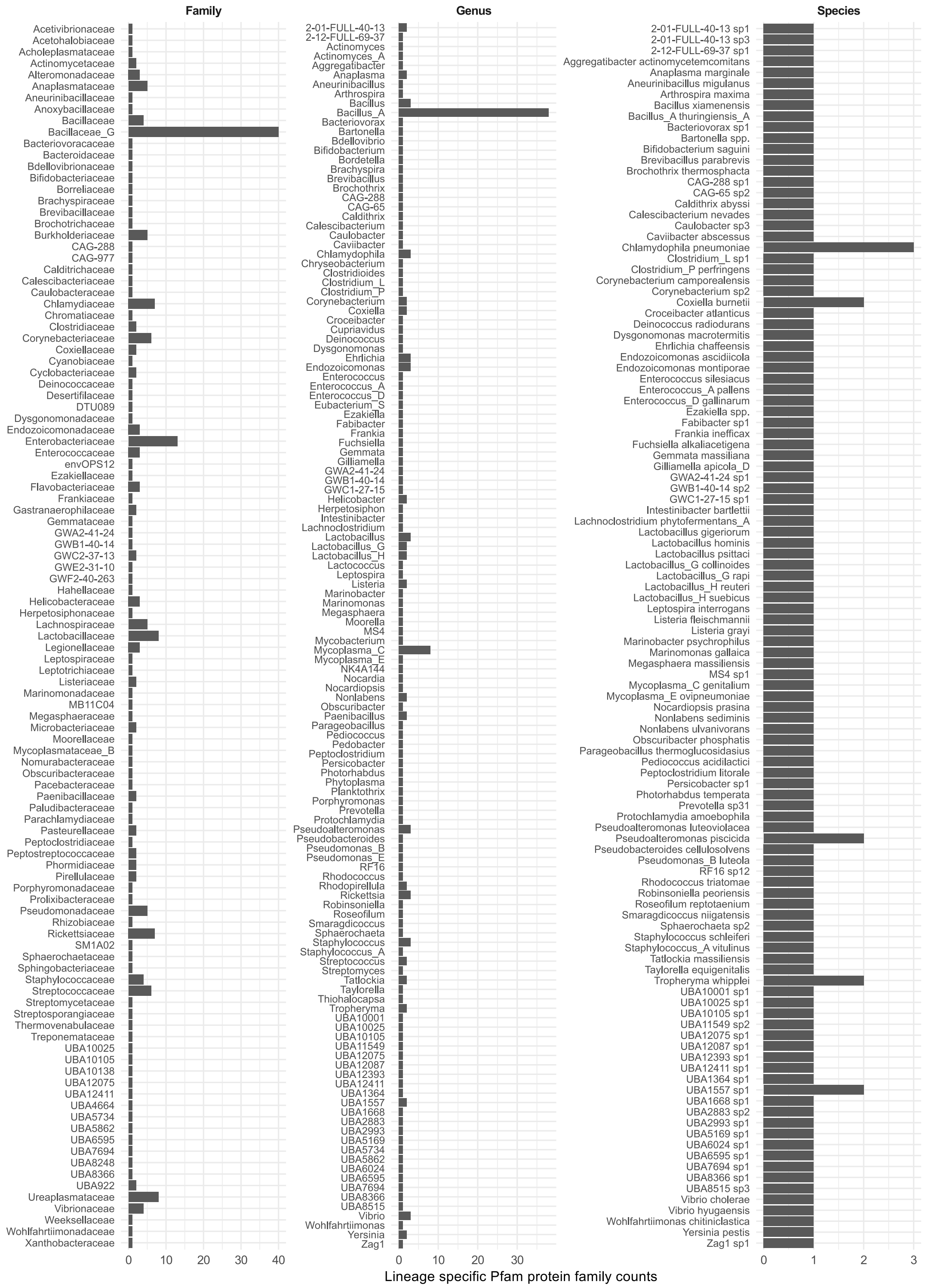

Supplemental Figure S7. Taxonomic distribution of lineage-specific Pfam protein families in lower levels. The taxonomic identity of each lineage-specific Pfam protein family was determined and counted at each level. Counts at higher levels include all lineage-specific traits at that level and all encompassing lower levels. See **Supplemental Figure 6** for Pfam protein family counts at the phylum, class, and order levels.

Reductive dehalogenase (PF13486)

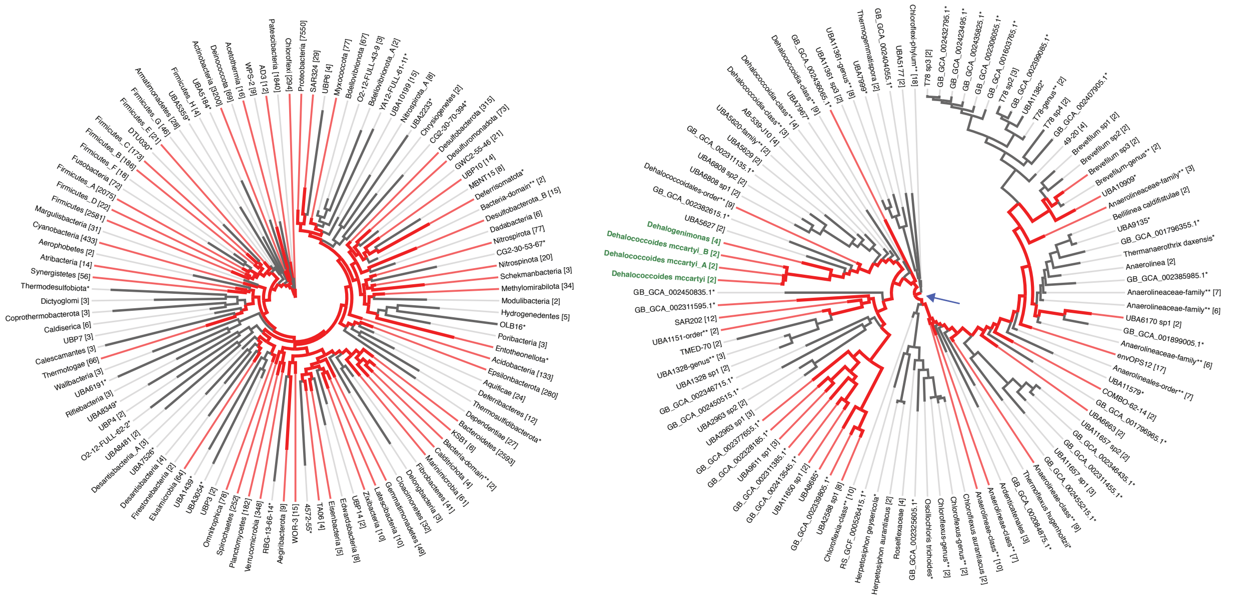

A: Phylum level

B: Phylum Chloroflexi, Species level

Supplemental Figure S8. Example AnnoTree search for reductive dehalogenases (Pfam PF13486) at the (A) phylum level and (B) Chloroflexi species level. In (B), the blue arrow points to the AnnoTree-derived hypothesis of an ancient origin for *rdhA* within the Chloroflexi, imputing gene loss rather than gene acquisition has been the dominant process in this phylum. Historically, the narrow distribution of this trait in the closely-related genera *Dehalococcoides* and *Dehalogenimonas* (green text) and the propensity for *rdh* genes to be within genomic islands or regions of significant genome rearrangements has led to conjecture that *rdh* genes and OHR may be acquired characteristics for these Chloroflexi (2, 3). AnnoTree's identification of a patchy distribution indicates further research is required to assess *rdh* evolutionary origins within the Chloroflexi.

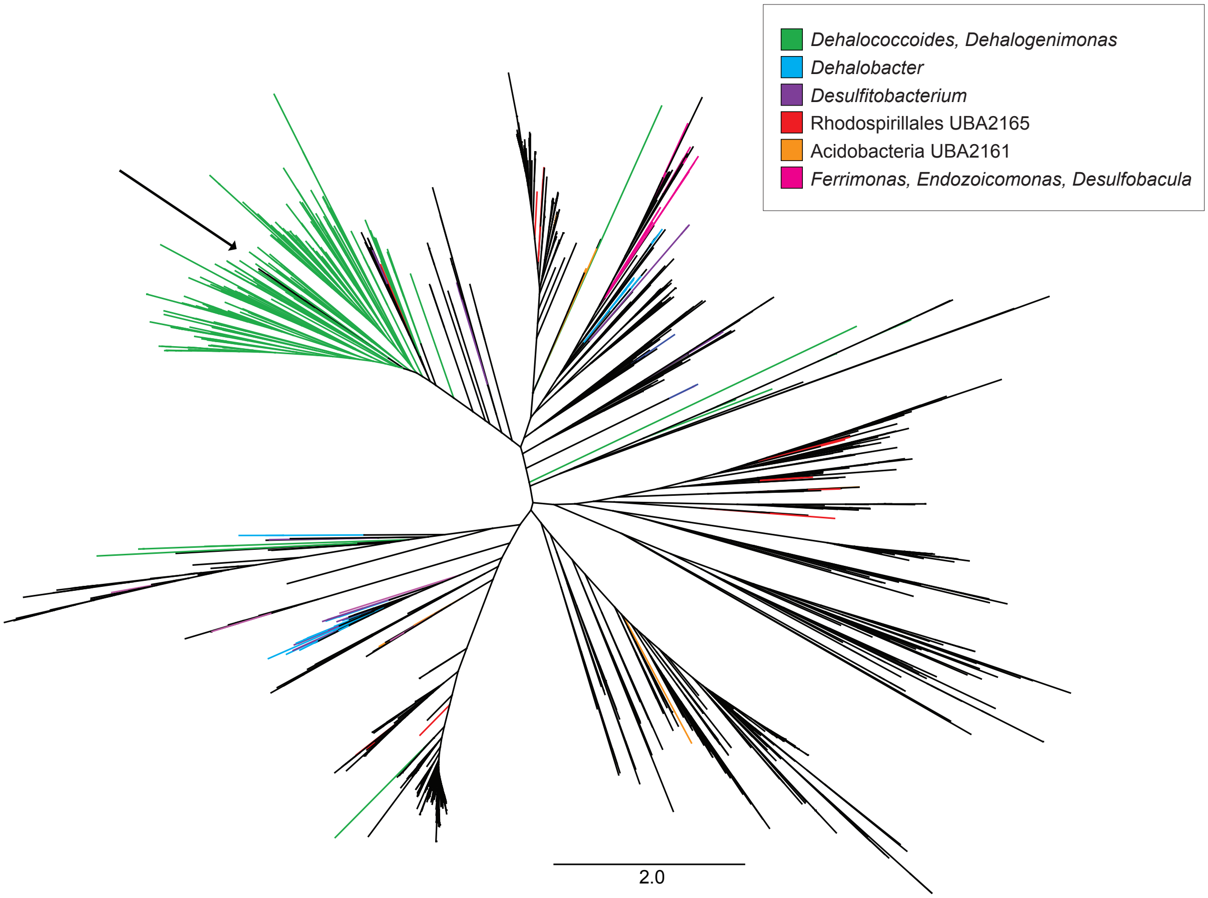

Supplemental Figure 9. Reductive dehalogenase diversity as identified by AnnoTree. The phylogenetic tree is based on a MUSCLE v. 3.8.1551 alignment (4) of the 1,299 RdhA sequences downloaded from an AnnoTree search of Pfam PF13486. The alignment was trimmed of all columns composed of >95% gaps, and the tree inferred using the RAxML implementation on the Cipres Science Gateway under the LG model and with bootstrapping automatically determined (5). Colors highlight RdhA from organisms with greater than 5 *rdhA* genes per genome, which represent known and putative obligate organohalide respirers. Green: *Dehalococcoides/Dehalogenimonas*, blue: *Dehalobacter*, purple: *Desulfitobacterium*. Two novel lineages are highlighted in red (Rhodospirillales UBA2165) and orange (Acidobacteria UBA2161). Organisms with >5 *rdhA* genes but no recorded organohalide respiration are highlighted in pink. The black arrow indicates a possible cross-phylum horizontal gene transfer event originating from the Chloroflexi.

Supplemental Tables

Supplemental Table S1. Comparison between the *rdhA* genes identified by AnnoTree, Pfam, and a curated *rdhA* database from 2013 (RDOG = reductive dehalogenase orthologous groups, Hug *et al.*(6)).

	# genes	# phyla	# genera
AnnoTree	1,299	38	385 (153 named)
Pfam (PF13486)	263	16	73
RDOG database	264	6	19

Supplemental Table S2. Summary statistics for organisms containing greater than 5 *rdhA* genes in their genomes. Previously uncharacterized organisms, representing novel potential obligate organohalide respirers, are highlighted in grey.

Organism	GTDB ID	metabolism	# rdh
Dehalococcoides mccartyi strain MB	RS_GCF_001459095.1	obligate OHR	33
Dehalococcoides sp. CBDB1	RS_GCF_000009025.1	obligate OHR	31
Dehalococcoides sp. UCH007	RS_GCF_001010485.1	obligate OHR	29
Dehalogenimonas alkenigignens	RS_GCF_001466665.1	obligate OHR	29
Dehalogenimonas lykanthroporepellens BL-DC-9	RS_GCF_000143165.1	obligate OHR	26
Dehalococcoides mccartyi CG5	RS_GCF_000830885.1	obligate OHR	26
Dehalogenimonas formicexedens	RS_GCF_001953175.1	obligate OHR	25
Dehalobacter restrictus DSM 9455	RS_GCF_000512895.1	obligate OHR	24
Dehalococcoides mccartyi CG3	RS_GCF_001889305.1	obligate OHR	24
Dehalogenimonas sp. WBC-2	GB_GCA_001005265.1	obligate OHR	21
Dehalococcoides mccartyi 195	RS_GCF_000011905.1	obligate OHR	18
Dehalobacter sp. DCA	RS_GCF_000305775.1	obligate OHR	17
Rhodospirillales UBA2165	GB_GCA_002327565.1	unknown	13
Acidobacteria UBA2161	GB_GCA_002328835.1	unknown	8
Ferrimonas sediminum	GB_GCA_900100175.1	flexible, no reported OHR	8
Endozoicomonas sp. S-B4-1U	RS_GCF_900174585.1	flexible, no reported OHR	8
Desulfitobacterium hafniense DCB-2	RS_GCF_000021925.1	flexible, OHR	7
Desulfitobacterium dehalogenans ATCC 51507	RS_GCF_000243155.2	flexible, OHR	7
Desulfobacula phenolica	GB_GCA_900105645.1	flexible, no reported OHR	6
Desulfitobacterium sp. PCE1	RS_GCF_000384015.1	flexible, OHR	6

Supplemental Data Files

Supplemental Data File 1. Lineage-specific KEGG and Pfam annotations and corresponding lineages
Supplemental Data File 2. KEGG and Pfam annotation phylogenetic distribution metrics
Supplemental Data File 3. KEGG pathway homoplasy metrics
Supplemental Data File 4. Taxon enrichment of homoplasic Pfam annotations
Supplemental Data File 5. Taxon enrichment of homoplasic KEGG annotations

Supplemental References

1. Parks,D.H., Rinke,C., Chuvochina,M., Chaumeil,P.A., Woodcroft,B.J., Evans,P.N., Hugenholtz,P. and Tyson,G.W. (2017) Recovery of nearly 8,000 metagenome-assembled genomes substantially expands the tree of life. *Nat. Microbiol.*, **2**, 1533–1542.
2. McMurdie,P.J., Behrens,S.F., Müller,J.A., Göke,J., Ritalahti,K.M., Wagner,R., Goltsman,E., Lapidus,A., Holmes,S., Löffler,F.E., *et al.* (2009) Localized plasticity in the streamlined genomes of vinyl chloride respiring Dehalococcoides. *PLoS Genet.*, **5**, e1000714.
3. McMurdie,P.J., Hug,L.A., Edwards,E.A., Holmes,S. and Spormann,A.M. (2011) Site-Specific Mobilization of Vinyl Chloride Respiration Islands by a Mechanism Common in Dehalococcoides. *BMC Genomics*, **12**, 287.
4. Edgar,R.C. (2004) MUSCLE: multiple sequence alignment with high accuracy and high throughput. *Nucleic Acids Res.*, **32**, 1792–7.
5. Stamatakis,A. (2014) RAxML version 8: a tool for phylogenetic analysis and post-analysis of large phylogenies. *Bioinformatics*, **30**, 1312–1313.
6. Hug,L.A., Maphosa,F., Leys,D., Löffler,F.E., Smidt,H., Edwards,E.A. and Adrian,L. (2013) Overview of organohalide-respiring bacteria and a proposal for a classification system for reductive dehalogenases. *Philos. Trans. R. Soc. B Biol. Sci.*, **368**.
